# Trends in the Engineering of Adeno-Associated Virus (AAV) for Precision Gene Delivery to the Central Nervous System (CNS)

**DOI:** 10.3390/ijms27135668

**Published:** 2026-06-23

**Authors:** Sola Oloruntimehin, Alexander Malogolovkin

**Affiliations:** Laboratory of Molecular Virology, E.I. Martsinovsky Institute of Medical Parasitology, Tropical and Vector-Borne Diseases, Clinical Center, I.M. Sechenov First Moscow State Medical University, Moscow 119991, Russia

**Keywords:** central nervous system, CNS, AAV, neurodegenerative, Parkinson’s disease, Alzheimer’s disease, spinal muscular atrophy, AADCD

## Abstract

Rare genetic disorders of the central nervous system (CNS) remain some of the most complex and challenging diseases to treat for several reasons. Targeting the CNS, especially the brain, presents one of the greatest obstacles in gene therapy using adeno-associated virus (AAV) vectors. Although various AAVs have been identified for their ability to transduce different cells in the CNS, their effectiveness and efficiency are significantly limited by the presence of neutralising antibodies (NAbs) and restricted cargo capacity. Despite these challenges, our understanding of AAV structure and technological advances continue to enable researchers to develop innovative strategies that have resulted in groundbreaking, FDA-approved therapeutic products now available for Leber congenital amaurosis (LCA) (Luxturna^®^), spinal muscular atrophy (SMA) (Zolgensma^®^), and the two recent gene therapy products for aromatic L-amino acid decarboxylase (AADC) deficiency, Kebilidi^®^ and Upstaza^®^, which currently hold FDA and EMA approval, respectively. This review aims to highlight recent advances in the field of AAV gene therapy for neurological disorders, identify research gaps, and suggest areas for future investigation to enable potential breakthroughs particularly in neurodegenerative, neurodevelopmental, and neuromuscular disorders. We foresee that more tissue- and cell-specific AAV vectors designed using AI-powered platforms will emerge to precisely and efficiently target specific brain regions, transforming how CNS disorders are treated.

## 1. Introduction

For millions, the simple act of holding a pen, sitting properly or recalling loved one’s name becomes a challenge as the system that coordinates these functions begins to fray. To others, these malfunctions start early in life due to genetic mutations. Neurological conditions account for some of the greatest medical diseases, many of which have effective long-term treatments imposing serious financial burdens. Disorders such as spinal muscular atrophy (SMA), aromatic L-amino acid decarboxylase (AADC) deficiency, Parkinson’s disease (PD), gangliosidoses (GM1 and GM2), mucopolysaccharidoses (MPSs), lysosomal storage diseases, and many others are marked by progressive neuronal dysfunction, degeneration, or biochemical imbalances that severely impact quality of life and reduce lifespan. Recently, it was estimated that 40% of the world population, totalling about 3.4 billion, had a condition affecting the central nervous system [1]. Traditional therapies, including enzyme replacement therapy (ERT), small molecules, and symptomatic management, have yielded limited success due to poor blood–brain barrier (BBB) penetration, temporary effects, or their inability to halt disease progression. Furthermore, these standard pharmacotherapies are often associated with side effects, lack disease-modifying activity, and do not prevent long-term neurological decline in diseases like Parkinson’s and Alzheimer’s [2]. These limitations underscore the need for a more targeted approach that will provide long-term, reliable therapeutic benefits to patients. Among the candidates that have shown the possibility of delivering effective therapy to the CNS is adeno-associated virus (AAV)-based gene therapy.

AAV vectors continue to gain attention as potential platforms for CNS-directed gene therapy, owing to their favourable safety profile, capacity for long-term transgene expression in post-mitotic neurons, and versatility across delivery routes (intravenous, intrathecal, intracisternal, and direct parenchymal injection). Natural virus serotypes, such as AAV9, AAVrh8, and AAVrh10, and engineered capsids (e.g., AAV-PHP.B, AAV-PHP.eB, and CAP-B10) display broad CNS tropism and the clinical potential to overcome the BBB, particularly in neonatal or infant patients [3,4]. Clinical translation of AAV therapy in the CNS is exemplified by the landmark approval of *onasemnogene abeparvovec* (Zolgensma^®^) for SMA, which demonstrated that systemic AAV9 delivery of SMN1 can reliably alter disease course and survival [5,6,7]. Similarly, *eladocagene exuparvovec* (Upstaza^®^), an AAV2-based therapy for AADC deficiency, established the feasibility of intraputaminal delivery of a dopamine biosynthetic enzyme gene to restore neurotransmitter function and improve motor development [8]. These clinical successes underscore the transformative potential of AAV vectors for neurogenetic disorders.

Additionally, a growing pipeline of investigational AAV programmes targets a diverse range of CNS disorders. For example, AAV-mediated delivery of dopamine-synthesising enzymes (TH, AADC, and GCH1) and neurotrophic factors for Parkinson’s disease has shown sustained transgene expression and motor improvements in early trials [9,10], though further optimisation of vectors and clinical endpoints remains necessary. Lysosomal storage disorders (LSDs) present some of the most debilitating behavioural and neuropsychiatric disorders in infants, although there are also juvenile manifestations [11,12]. In LSDs such as gangliosidoses (GM1 and GM2) and mucopolysacharidoses (MPS II, IIIA and C), AAV vectors are employed to restore lysosomal enzyme activity either via systemic AAV9 delivery or CNS-targeted injection routes (e.g., bi-thalamic rAAVrh8 for GM2) [13,14]. Early clinical studies have also reported biochemical correction (restored enzyme activity, reduced ganglioside or glycosaminoglycan accumulation) and signs of clinical stabilisation [13,15,16,17]. Recently, efforts have also been extended to rare neurotransmitter and metabolic disorders, including DRD, where strategies using AAV to deliver GCH1 or combinations of dopamine biosynthetic enzymes show strong preclinical promise [18,19]. All of these examples demonstrate the applicability of AAV gene therapy in both neurodevelopmental and neurodegenerative diseases.

Despite these landmark achievements, significant challenges still exist. Achieving efficient, targeted CNS transduction at clinically acceptable vector doses is hindered by the BBB, vector biodistribution, and pre-existing immunity to AAV capsids [20,21]. Safety concerns, such as hepatotoxicity, neuroinflammation, and dorsal root ganglia pathology in animal models, require careful dose selection, immune modulation, and long-term follow-up to properly evaluate the therapeutic potential of any AAV vector. Additionally, issues like the durability of expression, re-dosing constraints due to immunogenicity, production costs, and manufacturing scale-up pose other major translational obstacles.

Nevertheless, the convergence of capsid engineering, promoter optimisation, and new delivery methods, along with a rapidly growing body of preclinical and clinical data, positions AAV gene therapy at the forefront of a therapeutic revolution that will benefit patients with neurodegenerative disorders worldwide. As the field progresses, the challenges are not only to improve safety and efficacy but also to incorporate biomarker-driven outcome measures, equitable access strategies, and long-term follow-up to ensure lasting and global impact. For this review, we searched databases such as PubMed and Google Scholar for relevant articles with a focus on gene therapy clinical reports. Search keywords included ‘gene therapy’, ‘adeno-associated virus’ and/or ‘AAV’, ‘central nervous system’, ‘CNS disorders’, ‘Huntington’s disease’, Alzheimer’s disease’, ‘SMA’, and/or ‘clinical trial’. Where clinical data are not available, we consider experimental reports from open sources. In some cases, we also searched companies’ websites for clinical trial reports. Hence, we provide an overview of recent advances in adeno-associated virus (AAV)-mediated gene therapy for central nervous system (CNS) disorders, with particular emphasis on key clinical milestones, emerging mechanistic understanding, and critical knowledge gaps that must be addressed to realise the full therapeutic potential of these evolving modalities. We also offer a considered perspective on priority areas for future investigation that are likely to be essential for achieving durable and clinically meaningful outcomes.

## 2. Adeno-Associated Virus Vectors with Tropism to the CNS

**Figure 1:**
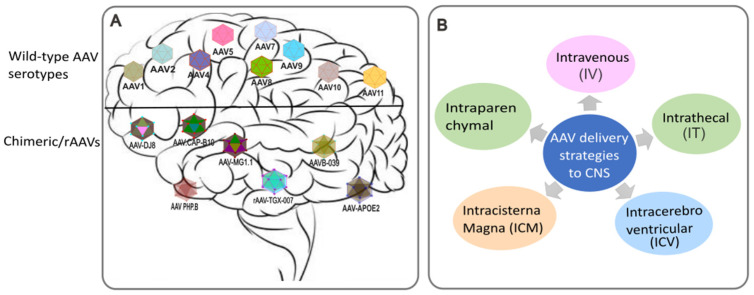
(**A**) Some notable wild-type (natural) and engineered AAV vectors that have demonstrated significant tropism in the central nervous system (represented by the brain). (**B**) Different injection routes to deliver AAV vectors into the brain and the CNS.

**Figure 2:**
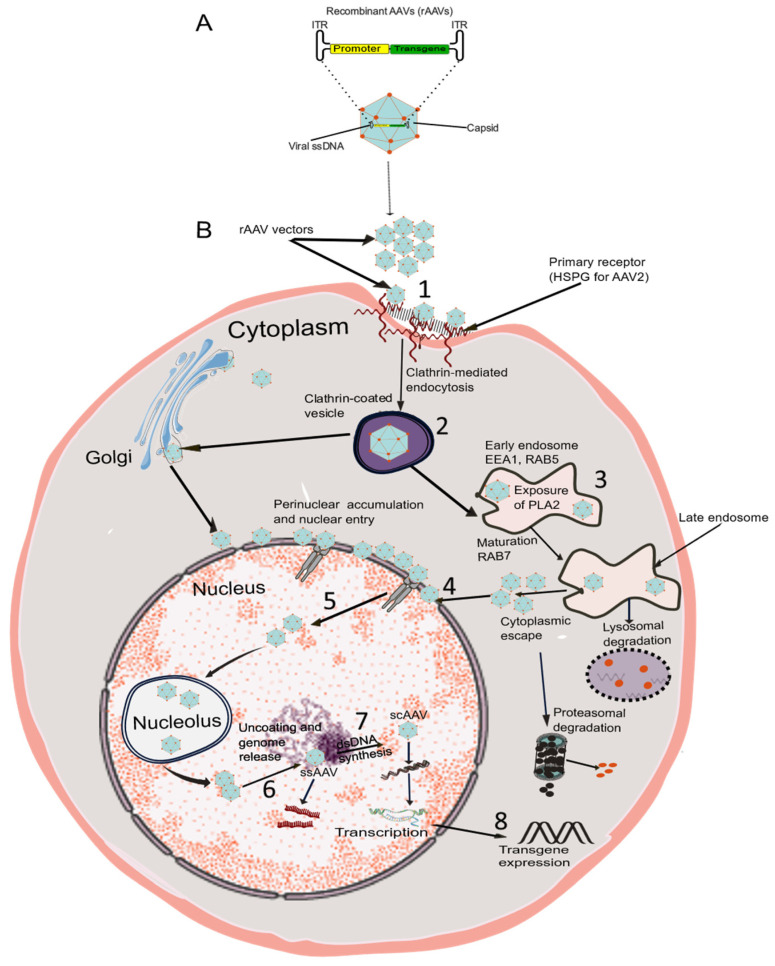
Schematic illustration of AAV transduction and nuclear trafficking. (**A**) Recombinant/engineered AAV vector with transgene and promoter. (**B**) (1) The initial step in the AAV transduction and intracellular trafficking is the binding of the AAV vector to both the primary receptors, like HSPG for AAV2 or LamR for AAV8, and other secondary receptors. (2) For cell entry, the viruses undergo clathrin-mediated endocytosis via clathrin-coated vesicles. (3) The formation of early and late endosomes and viral maturation. Mature viruses are then released from the endosome. (4) Released viruses form perinuclear accumulation and nuclear entry by interacting with importin-β and other karyopherins. (5) The viruses that escaped ubiquitination degradation enter the nucleolus as a temporary sequestration site that helps to facilitate viral uncoating. (6) Viral uncoating and genome release. (7) The crucial step of the dsDNA genome formation is facilitated by the host’s DNA polymerase. (8) The double-stranded AAV genome is transcribed and leads to eventual transgene expression.

AAV vectors continue to attract attention, emerging as the leading platform for gene delivery to the CNS, offering promise for treating various neurological disorders due to their favourable safety profile, long-term transgene expression, and ability to transduce non-dividing cells such as neurons [21,22,23]. They are non-enveloped viruses with an icosahedral capsid approximately 25 nm in diameter, composed of three viral proteins (VP1, VP2, and VP3) in a 1:1:10 ratio [21]. The capsids encapsulate a single-stranded DNA (ssDNA) genome of approximately 4.7 kb, flanked by inverted terminal repeats (ITRs) that serve as origins of replication and packaging signals [21,24]. For CNS applications, certain AAV serotypes demonstrate superior tropism and transduction efficiency. AAV9 and AAVrh10 are particularly notable for their ability to cross the BBB following systemic delivery, making them leading vectors for CNS gene delivery [25]. Other serotypes, including AAV1, AAV2, AAV5, AAV6, AAV8, and AAVrh8 (see Figure 1), also exhibit affinity for neural tissues but with varying cellular specificities within the CNS [25]. Each AAV serotype has a specific primary receptor that facilitates its transduction and cellular trafficking [26] (see Figure 2). The recent FDA approval of Kebilidi and EMA-backed Upstaza^®^ for the treatment of AADC deficiency demonstrates remarkable progress in the fight against rare genetic diseases in different areas (capsid engineering and novel delivery options). As clinical trials advance and vector design technologies evolve, AAV-based intervention may soon become a cornerstone of precision neurotherapeutics.

### 2.1. Advances in AAV Design for CNS Delivery

Ongoing efforts focus on improving the transduction efficiency of AAV vectors throughout the CNS, targeting different brain regions and cell types. Despite their small size and cargo capacity, AAV vectors continue to attract attention for several reasons: (i) the ability to modify AAV capsids to enhance their cell type or tissue specificity; (ii) combining these advanced capsids with modern gene regulatory elements, reporters, sensors, and effector cargos enables physiologically relevant transgene expression; (iii) they facilitate detailed understanding of how different brain regions respond to gene therapy. For comprehensive reviews of recent advances in AAV technology, including capsid modifications, regulatory elements, and functional cargos used to improve targeting and expression control, we direct readers to Challis et al. and Kolesnik et al. [27,28].

### 2.2. AAV Transduction and Intracellular Trafficking

The foundation of AAV vector engineering relies on a comprehensive understanding of the AAV pathway and cellular trafficking (see Figure 2). This host-factor-dependent process begins with attachment to abundant cell-surface glycans (e.g., heparan sulfate proteoglycan (HSPG) for AAV2, an uncharacterised type-I transmembrane protein (KIAA0319L) named AAV receptor (AAVR)) [29]. See Table 1 for other serotype-specific AAV receptors. Also, using genome-wide screening, several conserved host factors required for productive AAV transduction, such as GPR108 (a GPCR-family protein) [30], and CRISPR-identified TM9SF2 [31] modulating AAV uptake and trafficking have been identified. AAV binding to the cells is followed by endocytosis and early intracellular trafficking, often involving clathrin-coated pits, but alternative pathways (caveolar or micropinocytosis-like routes) have been reported for particular serotypes/cell types [32]. Acidification of early/late endosomes induces capsid rearrangements that expose internal regions of VP1/VP2 (the VP1u unique region) with a functional phospholipase A2 (PLA2) motif; this activity (and associated conformational changes) is important for destabilising endosomal membranes and enabling escape to the cytosol [33]. The mature capsids must then escape the endosome and undergo proteasomal degradation and ubiquitination. Only a minority of internalised particles escape productive endosomal compartments: the capsid sequence and host endosomal proteases. Enhancing escape (e.g., by modifying capsids or transiently inhibiting degradative pathways) improves transduction [34,35]. Then, AAV capsids interact with nuclear import machinery (e.g., importin-β and other karyopherins) and are thought to enter the nucleus either as intact particles through nuclear pores or after partial uncoating near the nuclear envelope. The transduction efficiency of AAV vectors can be modulated by manipulating nuclear import signals [36]. After nuclear entry, uncoating releases the single-stranded AAV genomes, which require host DNA polymerases to synthesise the complementary strand. This step is regarded as a rate-limiting step for many serotypes and/or cell types. The invention of self-complementary AAV (scAAV) vectors bypasses this bottleneck by packaging an inverted repeat genome that folds into double-stranded DNA, markedly accelerating transgene expression in non-dividing cells [37,38]. All of these fundamental understandings and discoveries in the AAV transduction pathway and intracellular trafficking are shaping the development and engineering of new and more efficient vectors.

Recently, Drouyer et al. developed customised AAV testing kits that contain mixtures of various AAV capsids tagged with unique barcodes for high-throughput next-generation sequencing (NGS) analysis. Their goal was to identify capsids that can effectively transduce human neural cells using human induced pluripotent stem cell (hiPSC)-derived brain organoids, as well as mouse and non-human primate (NHP) in vivo models after cerebrospinal fluid (CSF) delivery [42]. The study identified top-performing AAV variants, including AAV2.7m8, AAV-SYD12, AAV2-M1, and AAV2-L5, among others, which showed efficient transduction in human brain organoids. These variants exhibited specificity for neurons with minimal targeting of astrocytes. Validation in a mouse model confirmed efficient CNS transduction near injection sites, with AAV2-M1 demonstrating widespread brain distribution, including in cerebellar neurons. Such delivery methods are gaining traction because the intravenous injection route faces systemic barriers and complications [43,44,45]. This set of problems involves toxicity and mortality associated with high-dose systemic AAV administration [46]. Meta-analyses encompassing more than 250 clinical trials indicate that hepatotoxicity, thrombotic microangiopathy, and neurotoxicity constitute the predominant categories of serious treatment-related adverse events, with 11 deaths reported across eight trials and approximately one-third of studies documenting serious adverse events attributed to vector administration [47,48]. Intravenous AAV delivery, particularly at doses exceeding 1 × 10^14^ vector genomes per kilogram, frequently elicits dose-dependent hepatic injury, characterised by elevations in serum transaminases emerging 1 to 4 weeks post-infusion and, in the most severe instances, acute liver failure driven by immune-mediated sinusoidal endothelial cell injury and microthrombosis. Clinically, this has been underscored by fatal liver failure events following high-dose AAV gene therapy for muscular dystrophy, which have prompted regulatory investigations and renewed scrutiny of dose-escalation paradigms [46]. Collectively, these observations underscore the need for alternative delivery methods that would be more targeted while avoiding systemic injury and vector clearance. In Table 2, we present a comparison of performance metrics of natural capsids vs. engineered AAV vectors. These suggest the reasons why engineered vectors continue to attract more attention.

Recombinant AAV vectors such as AAV-DJ, AAV-DJB, AAV-PHP.B, AAV-PHP.eB, AAV-PHP.S, AAV-CAP-B10, AAV-CAP-Mac, AAV-MG1.1, AAV-MG1.2, and rAAV-TGX-007 continue to demonstrate improved transduction across major CNS cell types (neurons, astrocytes, oligodendrocytes, microglia, and endothelial) and brain regions (cortex, hippocampus, striatum, and cerebellum) [55,56,57,58,59,60]. The delivery encompasses all the routes of administration mentioned above. Figure 2 shows general intracellular trafficking of AAV vectors to protein synthesis.

A study on NHPs showed that AAV2-M1 and AAV-SYD12 achieved effective CNS transduction across multiple brain and spinal cord regions [42]. Although this approach is promising, several questions remain. The study focused on direct CNS delivery via CSF routes to bypass the BBB, but challenges persist for achieving effective systemic intravenous delivery capable of crossing the BBB without requiring high vector doses or causing off-target toxicity. While human brain organoids provide a valuable human-relevant system for testing AAV tropism and efficiency, they lack many in vivo biological complexities, such as the BBB, immune system interactions, and complete brain cytoarchitecture, which may limit their predictive accuracy for in vivo human CNS transduction. Additionally, even with CSF delivery, none of the AAV variants demonstrated deep parenchymal penetration of the brain in vivo, indicating a need to engineer vectors or delivery methods capable of wider CNS distribution. An additional concern is that rodent-selected AAV vectors often do not translate efficiently to NHPs or humans, as seen with AAV-PHP.B/PHP.eB. Although this study attempted to address this through multi-species screening, the use of only one NHP limits statistical robustness; larger NHP cohorts are necessary to confirm cross-species efficacy. Finally, it is important to consider how pre-existing immunity or neutralising antibodies may influence the effectiveness of these bioengineered capsids in clinical settings. The long-term expression stability, potential off-target effects, and safety profiles of these new AAV variants still require further clarification through preclinical and clinical studies.

### 2.3. Promoter Considerations in AAV Vector Design

Promoters are the “software” of AAV therapy because they determine what and how much therapeutic gene is expressed. Another area of interest in AAV vector design for CNS delivery has been the incorporation of various promoters. Different classes of promoters have been tested for AAV vector delivery with varying degrees of gene expression cascades and associated toxicities. Strong, ubiquitous, and constitutive promoters like CMV (human or murine cytomegalovirus immediate-early promoter), CAG/sCAG (CMV-enhancer + chicken β-actin promoter ± intron), EF1α (elongated factor 1 alpha), and CBh/CBA (chicken β-actin hybrid variants) are very good choices considering their strong, early expression capabilities [61,62,63]. However, studies have shown that CMV can be prone to transcriptional silencing in some tissues [64]. Besides the diminished durability of some of these promoters, another concern is the promoter–capsid interactions. Several studies have found that the same promoter may produce different expression patterns depending on the serotype/capsid (e.g., AAV9 vs. AAV1 vs. AAV2 vs. engineered capsids like PHP.eB) [65].

Some promoters are neuron-specific, such as hSyn (human synapsin I), which is widely used for pan-neuronal expression. The compact, neuron-restricted CaMKIIα promoter (calcium/calmodulin-dependent kinase II alpha) is considered to be strong in excitatory forebrain neurons. NSE (neuron-specific enolase) is another commonly used promoter for CNS delivery [66,67]. NSE is a ~1.8 kb regulatory element used in AAV vectors to drive strong, sustained, and specific transgene expression in mature neurons and neuroendocrine cells [68]. It is often considered for CNS gene delivery to avoid off-target expression in glia, but its large size limits the capacity for larger transgenes in AAV. GFAP (an astrocyte-specific promoter), MBP/MOG/PLP (oligodendrocyte lineage promoters) and Iba1/CD68 are glia-specific promoters, though not very common, but are also available for AAV vector design and delivery [69]. Pol III promoters, particularly for small RNAs such as U6 and H1 for shRNA and gRNA, respectively, are used for gene-editing or RNAi expression. However, they are not considered suitable for protein expression.

Recently, synthetic and engineered promoters, also called micro-promoters, have emerged. After reporting the creation of a hybrid form of CBA promoter (CBh) that demonstrated long-term and robust expression in many cells including motor neurons, Gray et al. also reported the development of 229 bp mouse methyl-CpG-binding protein-2 (MeCP2), which not only demonstrates neuron-specific expression but also allows an extra 570 bp packaging capacity in AAV compared to the 800 bp CMV promoter [62]. Similarly, Syn1-minCMV is another hybrid promoter designed for highly specific and robust gene expression in neurons. This promoter combines the neuron-specific regulatory elements of the human or rat synapsin I (SYN1) gene with minimal cytomegalovirus (CMV) core promoter (often ~50–60 bp). The Neuron-Restrictive Silencer Elements (NRSE/RE1) in the SYN1 promoter that bind to Neuron-Restrictive Silencer Factor (NRSF/REST) in non-neuronal cells particularly suppress expression outside the CNS [70]. Synthetic tissue-specific promoters and ultra-small micro-promoters (MP-84 and MP-135) have been developed to reduce cassette size while preserving strong expression [71]. These emerging promoters could be candidates of choice, especially considering the limited cargo capacity of AAV vectors.

## 3. AAV Gene Therapies in CNS Disorders

### 3.1. Neurodegenerative Disorders

Neurodegenerative disorders are conditions involving progressive degeneration of neurons and are often linked to toxic protein accumulation or impaired cellular clearance mechanisms. This loss of neurological functions results in some of the debilitating mental disorders affecting millions of people across the world today, with Alzheimer’s and Parkinson’s diseases presenting the most challenging pathological and clinical features.

#### 3.1.1. Alzheimer’s Disease (AD)

AD is considered one of the most challenging neurodegenerative disorders to treat. To date, no approved therapy can halt or reverse its progression. However, with advancements in engineering technology for AAV vector design, there is a gradual shift from conceptualisation to clinical application of AAV-dependent gene therapy targeting the underlying molecular drivers of AD, especially by leveraging the ability of recombinant AAVs (rAAVs) to deliver the transgene directly to the CNS for long-term expression. The genetic risk factor for AD is APOE ε4, and it is characterised by amyloid-β (Aβ) plaques, tau neurofibrillary tangles, neuroinflammation, synaptic dysfunction, and progressive neuronal loss. Following a landmark phase 1/2 clinical trial involving an intrathecal AAV therapy delivering APOE2 to APOE4 homozygous AD patients, and with incredible data showing detectable APOE2 in CSF, reductions in tau biomarkers (T-tau, P-tau) at 12 months, and a favourable safety profile with no serious adverse effects (NCT03634007), Yang et al. have recently identified some key gaps in APOE2 replacement strategy. They argued that it is unclear if APOE2 supplementation as a standalone therapy is sufficient to suppress APOE4-driven pathology in AD or if a co-repressor is needed to achieve sustained therapeutic benefits. Also, it is unclear whether introducing this therapy at the early or late stage of the disease will significantly improve therapeutic outcome. Hence, they developed an inducible AAV platform for APOE isoform replacement strategies, enabling temporary replacement of APOE4 with APOE2 in 5xFAD mice. Their data showed that long-term APOE2 expression with concurrent APOE4 silencing reversed Aβ deposition, glial activation, and cognitive decline, while short-term replacement potentially worsened pathology, linked to RAB24-mediated rebound adaptation affecting Aβ clearance and cholesterol metabolism [72]. This underscores the importance of timing and sustained expression in APOE-targeted therapy. Other notable approaches include neurotrophic factor delivery (AAV-BDNF) that aims to restore brain-derived neurotrophic factor in the entorhinal cortex and hippocampus, regions affected by AD, with a preclinical model demonstrating reversal of synaptic loss and neuroprotection [73,74], as well as the tau silencing approach, anti-amyloid gene therapy, and Klotho gene therapy. Currently, Voyager Therapeutics (VY1706) uses a BBB-penetrant TRACER^TM^ capsid to deliver a vectorised pri-amiRNA targeting all tau isoforms. In non-human primates, IV administration achieved 73% tau mRNA knockdown across brain regions. In mouse models, tauopathy treatment reduced pathological tau, preserved neurons, and improved function [75]. Also, the company is developing vectorised anti-amyloid antibodies for sustained CNS expression [76]. This approach could overcome the limitations of repeated monoclonal antibody infusions by providing continuous target engagement. KLTO-202, developed by Klotho Neurosciences, is an AAV-delivered form of the “anti-ageing” Klotho gene (s-KL). In preclinical AD models, Klotho protein has neuroprotective and cognitive-enhancing effects. Engineered capsids (AAVnerGene’s ATHENA platform) aim to improve CNS targeting and reduce liver tropism [77], which remains a major problem in AAV gene therapies. For details about other strategies and platforms for designing AAV vectors for effective CNS transduction, we refer readers to the article by Sharma et al. [40].

Furthermore, TREM2 (triggering receptor expressed on myeloid cells 2), a pattern-recognition receptor that is expressed almost exclusively by microglia in the central nervous system, where it regulates phagocytosis, lipid metabolism, chemotaxis, and cell survival [78,79], has been identified as a key driver in the “disease-associated microglia” (DAM) phenotype that clusters around amyloid-β (Aβ) plaques in AD models. Rare coding variants in TREM2, most notably R47H, particularly the p.R47H [80] variant as well as several other missense changes, are associated with a threefold increase in the risk of late-onset Alzheimer’s disease (AD) [81], yielding odds ratios similar to those conferred by the APOE ε4 allele [79,80]. Collectively, these studies support a causal role for microglia-driven innate immune processes in AD pathogenesis. In addition, loss-of-function mutations in TREM2 cause Nasu–Hakola disease [78,82], a presenile dementia accompanied by bone cysts, further highlighting the critical importance of TREM2 in preserving normal brain homeostasis.

Many phase 3 Alzheimer’s trials targeting amyloid (solanezumab (NCT02008357), gantenerumab, bapineuzumab, aducanumab ENGAGE/EMERGE, crenezumab (NCT01998841), etc.) have failed to show convincing clinical benefits despite target engagement [83,84]. Recurrent themes are: treatment started too late (mild–moderate dementia, when large-scale synaptic and neuronal loss is already present), heterogeneous populations (including amyloid-negative “Alzheimer’s” by clinical criteria), and modest effect sizes drowned out by variability and placebo response. In many cases, amyloid burden was reduced, but cognition did not improve meaningfully over 18–24 months [84], suggesting either that amyloid is an upstream trigger that must be treated pre-symptomatically, or that parallel pathologies (tau, inflammation, and vascular disease) dominate once dementia is established.

Despite these breakthroughs, there is a need to develop capsids with enhanced cell-type specificity, immune evasion capabilities, and equally offer transient immunosuppression as the use of magnetic resonance imaging (MRI)-dependent delivery continues to grow (NCT05040217 and NCT04120493) in clinical trials following the FDA approval of Kelibidi (eladocagene exuparvovec-tnaq) in 2024, which is administered directly to the brain. This method ensures accurate targeting, monitoring the distribution in real time, and overcoming the blood–brain barrier [85]. Another technology that will be very useful is the incorporation of PET reporter genes for dynamic tracking [86,87]. PET imaging allows for quantification, measurement of intensity and regional distribution of the expressed gene. Several PET reporter genes such as dopamine 2 receptor (D2R) [87,88], D2R mutant [89], type 2 cannabinoid receptor mutant, sodium iodide symporter, somatostatin receptor 2 [90,91] and herpes simplex virus type 1 thymidine kinase (HSV1-tk) [92,93] have been used. This method involves binding a reporter gene with a specific radiolabelled probe to measure the level of radioactivity, which directly reflects the gene expression level. This will help to evaluate the pharmacokinetics of AAV longitudinally beyond delivery monitoring [94].

#### 3.1.2. Parkinson’s Disease (PD)

Progressive loss of dopaminergic neurons in the substantia nigra is the main genetic deviation responsible for the development of PD. Although dopamine replacement therapies like levodopa (L-dopa) offer some relief symptomatically, they do not completely result in disease regression. Hence, researchers have focused on approaches that provide a long-term solution using AAV gene therapies.

One such approach is the attempt to restore GABAergic balance in patients [95]. MeiraGTx AAV-GAD therapy is based on this mechanism to deliver the glutamic acid decarboxylase (GAD) encoding gene to the subthalamic nucleus (STN), thereby improving local GABA production and rebalancing the circuitry of the basal ganglia. STN motor dysfunction is a hallmark of PD, and reducing hyperactivity in this region by the conversion of glutamate to GABA through GAD is a reliable approach. AAV-GAD is administered as a single-dose, minimally invasive intracerebral infusion delivered directly into the subthalamic nucleus (STN) [96]. The phase 2 clinical trial (NCT00643890), which included 45 PD patients, was a sham-controlled bilateral infusion. A high dose (2.1 × 10^11^ vector genomes) of AAV-GAD led to an 18-point improvement in Unified Parkinson’s Disease Rating Scale (UPDRS) Part III scores and an 8-point gain in PDQ-39 quality-of-life scores [97]. It will be worth following up to see the outcome of the trial (NCT05894343).

Furthermore, a recent NIH-sponsored clinical trial was designed to deliver a copy of the glial-cell-line-derived neurotrophic factor (AAV-GDNF). This open-label dose-escalation trial with convention-enhanced delivery to the putamen demonstrated safety and tolerability with long-term follow-up underway (NCT01621581).

Although AAV-GAD and AAV-GDNF remain the two primary approaches in efforts to restore motor neuron function in patients with PD, there has recently been a rise in other clinical techniques focusing on circuit-level reprogramming. For example, Chen et al. engineered AAV vectors with DREADDs (designer receptors exclusively activated by designer drugs) to selectively activate D1-MSNs in the striatum [98]. This method restored motor function in both mouse and primate PD models without causing dyskinesia [9,98]. In another study, Lee and his colleagues developed a self-complementary AAV vector encoding miR142-3p target sequences to suppress transgene expression in haematopoietic cells, thereby minimising peripheral toxicity [44]. Meanwhile, MeiraGTx is progressing its work on riboswitch-controlled AAV platforms that enable drug-inducible control of transgene expression, which could provide dynamic regulation and enhance safety, as previously mentioned.

**Figure 3:**
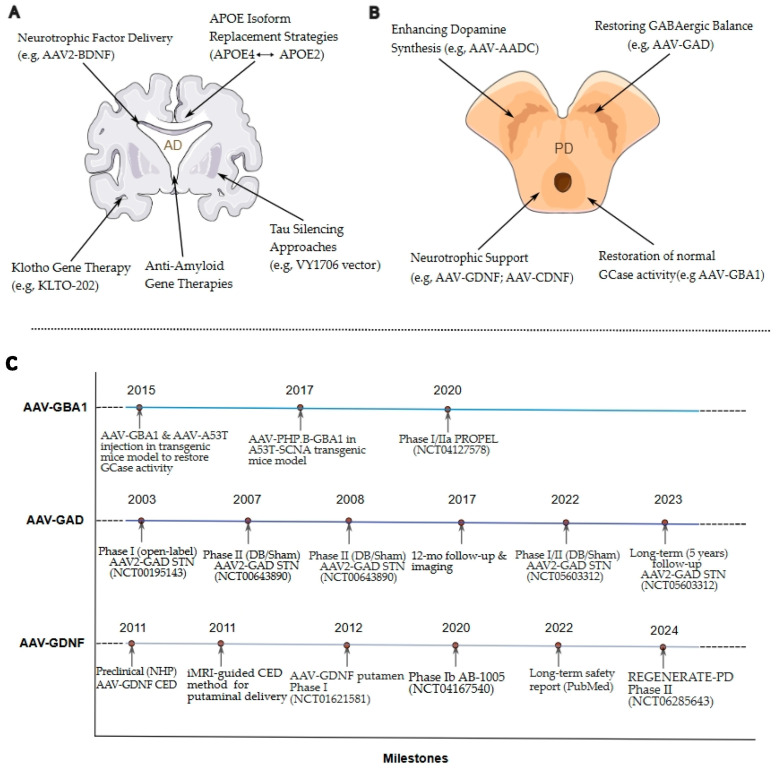
(**A**) Current clinical strategies aimed at treating AD (Alzheimer’s disease): neurotrophic factor delivery, APOE isoform replacement strategies replacing APOE4 with APOE2 in patients, approaches aimed at silencing tau protein, anti-amyloid gene therapy, and novel Klotho gene therapy approach; (**B**) PD (Parkinson’s disease): enhancing dopamine synthesis, restoring GABAergic balance, and neurotrophic support strategies. (**C**) Schematic illustration of the milestones in clinical translation of AAV-GAD, AAV-GDNF, and AAV-GBA1 therapeutic strategies for PD treatments, highlighting some important clinical trials [96,97,98,101,102]. STN—Subthalamic nucleus; CED—Convection-enhanced delivery; iMRI—interventional MRI, GAD—Glutamic acid decarboxylase; GCase- glucocerebrosidase.

Finally, in a large cohort study conducted by Sidransky et al. on Parkinson’s disease patients, it was discovered that a mutation in GBA1, the gene that encodes the lysosomal enzyme glucocerebrosidase (GCase) (primarily associated with the development of Gaucher disease), is also implicated in the development of PD [99]. According to one study, carriers of GBA1 variants have a potential risk of developing PD, with an odds ratio ranging from 2.2 to 30 [100]. Following some successes in restoring or normalising GCase pathway activities after injection of AAV-PHP.B-GBA1 into the A53 T-SCNA transgenic mouse model [101] or AAV-GBA1 and AAV-A53T injection into transgenic mice [102] (see Figure 3), Prevail Therapeutics has launched a phase 1/2a (NCT04127578), multicentre, open-label, ascending-dose PROPEL study to evaluate the safety of AAV-GBA1 (LY3884961) intracisternal administration to restore GCase activity and reverse PD. This opens a new frontier in the treatment of PD.

#### 3.1.3. Amyotrophic Lateral Sclerosis (ALS)

Amyotrophic lateral sclerosis (ALS) is a progressive, uniformly fatal, neurodegenerative disorder characterised by gradual loss of both upper and lower motor neurons, leading to muscle weakness, paralysis, and eventual respiratory failure with a very low survival rate (only 2–5 years post-diagnosis) [103,104]. While approximately 10% of cases are familial (fALS), linked to mutations in genes such as *SOD1*, *C9orf72*, *FUS*, and *TARDBP*, the majority (90%) are sporadic (sALS) with no known genetic cause [104]. SOD1 and C9orf72 are two of the most studied genetic causes of ALS, and they illustrate two different disease mechanisms [105,106]. SOD1 mutations cause a toxic gain-of-function: the mutant protein misfolds, aggregates, and disrupts multiple pathways, including proteostasis, axonal transport, mitochondrial function, lipid metabolism, and synaptic transmission [107]. These aggregates are a pathological hallmark of SOD1-familial ALS, and misfolded SOD1 inclusions are also found in some cases with other genetic mutations [106,107], suggesting that SOD1 misfolding can be a common downstream event. Clinically, SOD1 mutations account for roughly 10–20% of familial ALS and a smaller fraction of apparently sporadic cases, including de novo mutations, and motivated the development of SOD1-lowering antisense therapies. Contrariwise, the massive expansion of hexanucleotide repeats (GGGGCC) in the first intron of C9orf72 is the most commonly known genetic cause of ALS [105]. This expansion leads to three interacting mechanisms: haploinsufficiency of normal C9orf72 protein, accumulation of toxic RNA foci, and production of dipeptide repeat proteins through repeat-associated non-ATG (RAN) translation [108]. Masrori et al. recently showed that C9orf72 haploinsufficiency in microglia and astrocytes impairs endolysosomal pathways and blunts the normal transition of microglia into reactive, disease-associated states, altering neuroimmune responses in ALS [109].

Despite the heterogeneity in the disease development, 97% of all ALS cases are said to share common pathological hallmarks: the cytoplasmic mislocalisation and aggregation of TAR DNA-binding protein 43 (TDP-43) [110]. This proteinopathy disrupts RNA metabolism, promotes neuroinflammation, and ultimately drives motor neuron death [110,111]. The current FDA-approved therapies, riluzole, edavarone, and tofersen (for SOD1-ALS), offer only modest benefits, underscoring the urgent need for targeted, disease-modifying treatment like AAV-based gene therapy due to their ability to cross the blood–brain barrier and transduce the cells of the CNS.

New technologies are emerging to target ALS. Researchers at the University of Pennsylvania and CHOP (Children’s Hospital of Philadelphia) recently developed a peptide-modifying AAV9 (PM-AAV9) to deliver RNA interference (RNAi) for gene silencing (for example, miRNAs targeting *ATXN2* or *SOD1*), which demonstrates superior transduction in motor cortex layer V and spinal cord anterior horn cells compared to wild-type AAV9 and AAV-PHP.eB [110]. This capsid variant achieved 54% knockdown of target genes in the frontal cortex and 23–25% knockdown of brainstem and spinal regions after intracerebroventricular (ICV) injection in mice [110]. The TDP-43 mouse models showed prolonged survival by 54%, improved motor function (rotarod performance and gait scores), reduced neuroinflammation, and evidence of rescued transcriptomic dysregulation in >450 genes linked to ALS pathways [110]. The translation of these results to NHPs or human trials will be valuable to determine the prospect of this technology. Also, the use of neuroprotective genes, for example *TRIM72* (Tripartite Motif Protein 72), to counteract oxidative stress and mitochondrial dysfunction has been reported [112]. SinuaGene’s therapy named SNUG01 that delivers TRIM72 for ALS treatment has been granted Orphan Drug Designation (ODD) by the FDA according to the company’s report (https://www.sineugene.com/). AMT-162 by uniQure, an AAV5-based therapy encoding miRNA against SOD1, is currently in phase I/II trials (NCT06100276), with no safety concerns reported yet in the first cohort.

#### 3.1.4. Prion Diseases

Prion diseases, which include Creutzfeldt–Jakob disease (CJD), variant CJD, Gerstmann–Sträussler–Scheinker syndrome (GSS), fatal familial insomnia (FFI), iatrogenic CJD (iCJD), and other variants, are caused by the accumulation of misfolded prion protein (PrP) encoded by the *PRNP* gene, eventually leading to neurodegeneration of the brain [113,114]. This group of rare, progressive, and fatal neurodegenerative diseases are also called transmissible spongiform encephalopathies (TSEs) and affect both humans and animals. The accumulation of the abnormally folded protein (PrP^Sc^) causes neuronal dysfunction and cellular death [115]. Unlike other pathogens, prions contain no nucleic acid (DNA or RNA), which makes treatment attempts even more challenging. Recent research has identified specific intermediate conformations that drive this misfolding process, particularly involving structural changes in the α-helix H1 region and the highly conserved TVTTTT motif [116], an understanding that is crucial for tackling the disease. Current treatments are limited, and there is a substantial need for innovative therapeutic approaches. To date, the most advanced clinical trial for prion disease is Ionis Pharmaceuticals’ ION717 (PrProfile study), a non-AAV-based trial which completed enrolment in December 2024. This Phase 1/2 trial (NCT06153966) enrolled 56 symptomatic prion disease patients across 16 global sites. The trial uses intrathecal delivery of antisense oligonucleotides to reduce PrP mRNA. While this is promising, the therapy focuses on safety rather than efficacy. The need for repeated intrathecal dosing will be too burdensome for patients and families.

The therapeutic approach for prion diseases using AAV gene therapy centres on reducing or eliminating prion protein (PrP) expression. There are currently four significant AAV-related approaches targeting prion diseases. These strategies are based on the compelling evidence that PrP reduction halts disease progression in animal models [114,117,118]. The most advanced AAV-based approach involves the Coupled Histone tail for Autoinhibition Release of Methyltransferase (CHARM) system developed by researchers at the Broad Institute and Whitehead Institute. CHARM is a compact epigenetic editor that uses a histone H3 tail-Dnmt3l fusion to recruit and activate endogenous DNA methyltransferases. The strategy focuses on programmable DNA methylation to silence the *PRNP* gene. It was packaged into AAV vectors with zinc finger proteins (ZFPs) or other DNA-binding domains and demonstrates a 70–90% reduction in *PRNP* transcripts and a 60–80% reduction in *PRNP* protein levels in mouse brains [118]. The second method focuses on base editing technologies. Recent research has demonstrated AAV-delivered base editing systems that create stop codons in the *PRNP* gene using BE3.9max base editor. This experimental work showed 37% edit frequency at the target site, a 50% reduction in PrP levels, and a 52% increase in survival in humanised *PRNP* mice infected with pathogenic prions [117]. Furthermore, Sangamo Therapeutics has developed zinc finger repressor approaches that bind to *PRNP* DNA and shut down gene expression. The AAV-ZFR system showed dose-dependent PrP reduction up to 50% in C57BL/6 mice, extended survival from median 170 days to up to 494 days in prion-infected mice, and up to 95% PRNP repression in key brain areas like the hippocampus, thalamus, and putamen [119]. The new STAC-BBB capsid platform successfully delivered the vectors to 35 brain regions in non-human primates (NHPs) [120]. While these are laudable advancement in the treatment of prion diseases, it would worth following up to see how these translate into human clinical trials and, eventually, therapeutic treatment because of the BBB problem that may limit the effectiveness of these approaches. Also, there is the issue that the high dose requirement of (1 × 10^14^ vector genomes/kg) raises safety concerns and potential immune response.

### 3.2. Neurodevelopmental and Metabolic Disorder

#### 3.2.1. Aromatic L-Amino Acid Decarboxylase Deficiency (AADCD)

The recent FDA approval of KEBILIDI^TM^ (Eladocagene Exuparvovec) in November 2024 as the first AAV-based gene therapy for AADCD [121] is a milestone achievement for paediatric neurological disorders and CNS gene therapy. The technology behind Kebilidi^®^ involves the use of recombinant AAV2 (rAAV2) encoding the functional human *DDC* gene (which encodes the AADC enzyme) infused directly into the putamen via stereotactic neurosurgery. The SmartFlow Neuro Cannula has received FDA approval for precise intracerebral delivery of Kebilidi^®^ [121]. The open-label study involved 13 children with genetically confirmed AADCD who had no gross motor function at baseline. At 48 weeks post-treatment, 66.66% (8 out of 12) evaluable patients showed motor improvement, including head control, sitting, and standing. Dopamine production was restored, leading to improved mood, movement, and energy levels. One of the successfully treated children was a 3-year-old girl at Texas Children’s Hospital who was reported to show early signs of increased vitality and reduced oculogyric crises [122].

Several other AAV-based AADC gene transfer programmes beyond Kebilidi^®^/Upstaza are (or have been) in clinical trials, with significant progress towards addressing the debilitating deficiency. Midbrain-targeted AAV2-hAADC (NCT02852213), unlike Kebilidi^®^, which delivers functional gene to the putamen, infuses *hAADC* directly into the midbrain dopaminergic nuclei (bilateral substantia nigra pars compacta and ventral tegmental area) using intraoperative MRI-guided conventional-enhanced delivery (CED). This was aimed at restoring AADC, where it is normally expressed, so rescued neurons can supply dopamine along intact nigrostriatal projections [8]. Two cohorts of children aged 4–9 years old with genetically confirmed AADC deficiency received ~1.3 × 10^11^ vector genomes or 4.2 × 10^11^ vector genomes total; intraoperative gadolinium co-infusion documented ~98% SN and ~70% VTA coverage on MRI in aggregate analyses with remarkable results, showing resolution of oculogyric crises in six out of seven children by three months post-treatment, increased FDOPA uptake in midbrain/striatal targets, and motor skill gains through 12–24 months [8].

#### 3.2.2. Rett Syndrome

Rett Syndrome is a debilitating X-linked neurodevelopmental disorder caused primarily by mutations in the *MECP2* gene. This gene encodes a transcriptional regulator critical for neuronal maturation and synaptic plasticity. The disease leads to severe motor, cognitive, and autonomic dysfunction in children between 6 and 18 months of age.

The REVEAL phase 1/2 trial in adolescent, adult, and paediatric patients across the US, Canada, and the UK with TSHA-102 (Taysha Gene Therapy), which delivers a shortened MECP2 “mini-gene” using AAV9 via intrathecal injection, was shown to improve motor skills, communication, autonomic function, and seizure activity as early as 4 weeks after injection. While Taysha delivered a MECP2 mini-gene, their counterpart, Neurogene, in their trial designated NGN-401, delivered a full-length MECP2 gene. The vector encoding the gene was administered as a single injection into the cerebral ventricles, the fluid-filled space deep within the brain, to promote wider distribution across the brain. Both trials incorporated biological switches, miRARE and EXACT, respectively, that help regulate MECP2 protein production, since overproduction has negative effects. According to available data and an ongoing clinical trial (NCT05898620), the therapy is administered to female children, raising questions about whether it will have the same effects in male children.

### 3.3. Neuromuscular and Motor Neuron Diseases

#### Spinal Muscular Atrophy (SMA)

Spinal muscular atrophy (SMA) is a group of recessive neuromuscular disorders caused by mutations in the survival motor neuron 1 (*SMN1*) gene. It mainly affects nerve cells that control motor neurons, leading to progressive muscle loss and weakness due to wasting [123,124]. Most SMA types impact muscles involved in walking, arm movement, sitting, and head control. However, the disease progression in some types can cause difficulty in breathing and swallowing [125]. Importantly, SMA is considered a multisystem disorder affecting organs beyond the nervous system, including the heart, liver, kidneys, and pancreas [126,127]. The treatment options for SMA have changed significantly, with three approved therapies: nusinersen (antisense oligonucleotide SMN2 splicing modulator), risdiplam (oral SMN2 splicing modifier), and onasemnogene abeparvovec (AAV9-based *SMN1* gene replacement therapy, marketed as Zolgensma^®^) [128,129]. While nusinersen and risdiplam need to be administered multiple times, *onasemnogene abeparvovec* offers the benefit of a single dose but has safety concerns, especially hepatotoxicity and dose-limiting toxicities, although it could be mitigated with prophylactic prednisolone [130].

Recent research has centred on optimising promoter systems to achieve tissue-preferential expression without suppressing essential peripheral expression, addressing the multisystem nature of SMA. Song and colleagues developed EXG001-307, an AAV9-based vector that incorporates a hybrid neuron-preferential promoter combining synapsin and cytomegalovirus (CMV) regulatory elements. This design takes advantage of the neuronal specificity of synapsin, while the CMV enhancer provides enough peripheral expression to address non-neuronal disease manifestations as mentioned previously. In preclinical studies, EXG001-307 showed notable safety benefits, allowing administration at doses up to 6 × 10^14^ vector genomes/kg without treatment-related mortality, which is a considerable improvement over benchmark vectors that cause significant toxicity at lower doses. At 4.0 × 10^14^ vector genomes/kg, animals treated with EXG001-307 achieved a median survival of 191 days, compared to 32.5 days in animals receiving the benchmark vector. Tissue analysis of expression revealed reduced SMN levels in the liver and heart, while maintaining therapeutic levels in the brain and spinal cord, aligning with the intended preferential targeting [131,132].

A groundbreaking advancement in vector technology is the development of covalently closed-end AAV (cceAAV) vectors, which are part of a new generation of self-complementary AAV vectors that do not rely on mutant inverted terminal repeats (ITRs) for production. Unlike traditional scAAVs, which often contain incomplete genomes, cceAAV vectors consistently package more intact genomes. NKG-001, a cceAAV-based therapy, shares the same genetic sequence as onasemnogene abeparvovec except at the terminal level, where the mITR is replaced by TeIN, leading to enhanced transduction efficiency and transgene expression. Early clinical data from two children with SMA (aged one and two years) who previously received RNA-splicing-modifying drugs (nusinersen or risdiplam) show that NKG-001 was administered intravenously at relatively high doses (6 × 10^13^ to 1.2 × 10^14^ vector genomes/kg). The report indicates that the treatment was well-tolerated with no serious side effects, and both children experienced rapid motor function improvements. This vector technology addresses key manufacturing challenges associated with conventional scAAV vectors and may provide increased consistency and potency. However, despite promising results, the small sample size limits the ability to generalise findings or draw definitive conclusions. Larger, controlled trials are needed to validate these results. Additionally, the study involved older infants (12–24 months) who had already been treated with RNA-splicing modifiers, leaving uncertainty about the vector’s performance in treatment-naïve patients, older children, or adults, and whether gene therapy offers benefits beyond existing treatments.

Other clinical trials that have shown remarkable results include OAV101 IT (NCT05089656), which demonstrated a 2.39-point HFMSE improvement vs. 0.51 with placebo [133], GC101 (NCT05824169, NCT05901987, and NCT06421831) for SMA types 1, 2, and 3, which has entered phase 3, and LT01-101 by Lantu Biopharma, which is currently on phase 1/2. For detailed information about ongoing clinical trials, see Table 3. For a detailed review of the treatment of neuromuscular disorders with gene therapy and the target genes, we refer our readers to Wu and colleagues [134].

### 3.4. Lysosomal Storage Disorder

#### 3.4.1. Neuronal Ceroid Lipofuscinosis (Batten Disease)

Neuronal ceroid lipofuscinoses (NCLs), commonly known as Batten disease, are a group of serious inherited lysosomal storage disorders that primarily affect the nervous system. They result from mutations in various CLN genes (such as CLN2, CLN3, and CLN6), leading to defective lysosomal enzymes or membrane proteins. These defects cause progressive vision loss, seizures, deterioration of motor and cognitive functions, and early death. Although Cerliponase alfa (BioMarin Pharmaceuticals Inc., CA, USA), a recombinant proenzyme administered via intracerebroventricular (ICV) infusion every two weeks, is reported to significantly slow neurological decline and improve survival in children, it does not impact retinal dystrophy.

In early 2024, Henderson and his colleagues reported the first-in-human clinical trial of RGX-381 (AAV9.CB7.hCLN2), delivering human CLN2 via subretinal injection to treat Batten-associated retinopathy. The results of the phase 1/2 showed robust expression of tripeptidyl peptidase 1 (TPP1) enzyme in aqueous humour and preservation of photoreceptor structure at day 90 post-treatment [135]. AAV2-CLN2 CNS delivery showed dramatically extended survival in a subset of treated children up to 23.4 years in comparison to the typical 8–12 years [136]. This suggests that age at treatment and cranial volume may influence therapeutic efficacy. Furthermore, Amicus Therapeutics has launched two clinical trials for juvenile NCL (CLN3 disease) and CLN6 named AT-GTX-502 and AAV-CLN6, respectively. Both therapies are aimed at delivering functional *CLN3* and *CLN6* genes intrathecally. In a phase 1/2 study (NCT03770572), children treated with AT-GTX-502 showed stabilisation in physical impairment scores over 15 months, compared to an expected decline [137]. Similarly, 87.5% (seven out of eight) children who participated in the AAV-CLN6 clinical trial (NCT02725580) were reported to show stabilisation or improvement in motor and language scores over 24 months [138,139].

#### 3.4.2. Mucopolysaccharidoses

Mucopolysaccharidoses (MPSs) represent a group of 11 inherited lysosomal storage disorders caused by deficiencies in enzymes required for glycosaminoglycan (GAG) degradation, leading to progressive accumulation of undegraded substrates in multiple tissues [140]. The majority of MPS disorders (MPS I, MPS IIIA-D, MPS VII, and MPS IX) are autosomal recessive, while MPS II is X-linked recessive. These conditions manifest with multisystem involvement, including hepatosplenomegaly, skeletal dysplasia, cardiorespiratory complications, and in many cases, progressive neurodegeneration [140]. There are two traditional treatment options available for MPSs: enzyme replacement therapy (ERT) and haematopoietic stem cell transplantation (HSCT). While ERT has shown efficacy for somatic symptoms, its effectiveness is limited by its inability to cross the BBB, thus failing to address neurological manifestations. The drawbacks of these therapies call for alternative and more effective therapies like AAV gene therapy and a precise injection route that will target the disease’s pathological basis. Several AAV programmes for MPSs have progressed to human trials, with IV scAAV9 approaches (for early infants) and CNS-directed strategies (IT/ICT or intraparenchymal) being the most prominent [141,142].

Although AAV9 and AAVrh10 have consistently demonstrated efficient BBB crossing across different species compared to other natural variants (see Table 1), engineered capsids represent the next frontier in AAV technology, with AI-assisted design enabling enhanced tissue specificity, BBB crossing, and reduced immunogenicity. In MPS gene therapy, more attention has been given to the delivery strategy. For MPSs with primary neurological involvement (MPSs I, II, III, and VII), direct intracisternal delivery strategies have been developed: REGENXBIO recently announced their innovations for MPSs, RGX-111 (for MPSIIIA) and RGX-121 (for MPS II), which are both AA9-based therapies [143,144]. Both therapies are in phase I/II trials, and RGX-121 was initially considered for priority review. RGX-111 delivers a functional copy of the α-L-iduronidase (IDUA) gene to the CNS. The clinical trial (NCT03580083) enrolled 21 participants with two dose (1 × 10^10^ GC/g brain mass and 5 × 10^10^ GC/g brain mass) cohorts, which were administered using an investigational device by intracisternal or intracerebroventricular injection. For RGX-121 therapy on the other hand, there are two studies (NCT803566043 and NCT04571970) investigating the tolerability of the therapy in patients with the severe form of MPS II in children from 4 months to 5 years, with central nervous system involvement, and patients with severe Hunter syndrome aged 5 to 17, respectively [145]. However, a recent update on clinicaltrials.gov showed that these trials have been suspended following a single case of neoplasm (intraventricular CNS tumour) in a participant treated in a phase I/II study (NCT03580083) four years earlier.

Following pre-clinical successes in mouse models [146,147], Ultragenyx has recently completed a phase III clinical trial (NCT02716246) for UX111 (scAAV9 for MPS IIIA, also known as Sanfilippo syndrome), which uses IV delivery. The clinical data showed over 50% median reduction in CSF heparan sulfate levels (*p* < 0.0001) in the group of all patients who received the study’s high dose (3 × 10^13^ vector genomes/kg, n = 17), and improved neurodevelopmental outcomes [148]. However, the clinical trial’s data were met with the FDA’s Complete Response Letter (CRL) (BL 125845/0), citing specific chemistry, manufacturing, and controls (CMC)-related observations that are resolvable [149]. Moreover, we believe that there is a need for extended post-treatment follow-up beyond 34 months to properly evaluate the long-term effectiveness of the therapy and possible side effects, especially for patients who received high doses.

Rossi and colleagues recently reported their findings from a clinical trial of liver-directed gene therapy using AAV2/8 vectors to deliver the arylsulfatase B (ARSB) gene (AAV2/8.TBG.hARSB) in patients with mucopolysaccharidosis type VI (MPS VI) under a liver-specific promoter. In patients who receive a high dose (6.0 × 10^12^ vector genomes/kg), there is evidence of sustained serum ARSB enzyme activity at 38–67% of the mean healthy values after gene therapy, with no significant changes observed in endurance, cardiac, or pulmonary functions across patients. Overall, the treatment was well tolerated and suggested disease stabilisation with multi-year enzyme expression after a single gene therapy dose. However, the small patient cohort involved in the trial limits broad conclusions about efficacy and safety. Additionally, despite enzyme expression, some patients still had increased urinary GAG levels, similar to a previous report by Brunetti-Pierri et al., indicating incomplete biochemical correction or variable therapeutic response. Moreover, it is unclear whether the therapy will benefit naïve MPS patients who have no prior exposure to ERT or any other therapy, since the participants are those who discontinued ERT therapy. Finally, there is a need to clarify the impact of age at treatment and disease severity on therapeutic benefit, as the trial cohort had been exposed to traditional treatments.

#### 3.4.3. Gangliosidoses

Gangliosidoses are a group of inherited lysosomal storage disorders characterised by the accumulation of gangliosides in neural tissues due to deficiencies in specific lysosomal enzymes. The two main categories are GM1 gangliosidosis (caused by mutations on the *GLB1* gene encoding β-galactosidase) and GM2 gangliosidosis (including Tay–Sachs and Sandhoff diseases resulting from deficiencies in hexosaminidase A) [150]. These progressively devastating and ultimately fatal disorders currently have no approved disease-modifying treatments [13,150].

Currently, researchers are exploring various AAV serotypes with different tropisms for CNS targeting. AAV9 and AAVrh8 have demonstrated particularly favourable properties for gangliosidosis therapy: while AAV9 shows efficient BBB crossing capabilities when administered systemically, enabling widespread CNS transduction [151], AAVrh8 on the other hand demonstrates excellent distribution through CSF and axonal transport when delivered intracranially [13]. These recent advances include the development of dual-vector approaches for GM2 gangliosidosis, where separate AAVrh8 vectors deliver *HEXA* and *HEXB* genes simultaneously to restore hexosaminidase A function [13]. A phase 1/2 clinical trial (NCT04669535) investigating dual AAVrh8 vectors encoding HEXA and HEXB genes demonstrated promising results, with CSF HexA activity reaching ~13% of normal activity (0.59 nmol/h/mL) with peak effects at 12 weeks [13]. The CSF GM2 levels were also reported to decrease by up to 52.5% in four out of six infant patients, improved oral feeding ability (beyond 25 months), delayed seizure onset, and reduced seizure severity [152]. However, the therapeutic effects showed a decline by 24 weeks post-treatment, according to the report [13], indicating potential durability challenges.

For GM1 gangliosidosis, research has progressed along two main directions: one is the systemic delivery of AAV9 in mice, which has shown partial restoration of β-galactosidase activity in the CNS, 36–76% reduction in brain GM1-ganglioside content, and significantly extended lifespan (316–576 vs. 250–264 days in control) [151]. A recent clinical trial in type II GM1 patients (NCT03952637) involving a single infusion of AAV9 encoding β-galactosidase was well tolerated with increased CSF β-galactosidase and decreased GM1 ganglioside in all patients, stabilisation on Vineland Expressive Communication and Gross Motor domains, and improved myelination on MRIO and reduced cerebral atrophy [153].

However, several complications were reported in the clinical trial data. In both GM1 (NCT03952637) and GM2 (NCT04669535) trials, there was an occurrence of elevated liver enzymes, but they were manageable with corticosteroid regimens. The surgical procedure for GM2 resulted in wound dehiscence following intracranial delivery, although this was addressed through protocol modifications. Another concerning report was the differential responses observed in the GM2 trial. Juvenile patients with GM2 experienced worsening dystonia, leading to their exclusion from ongoing trials. The worsening dystonia in juvenile GM2 patients calls for a mechanistic understanding and tailored approaches. Despite detailed characterisation of immune responses indicating immunosuppression, immune reactions limit efficacy and require better mitigation strategies. For example, there is evidence of neutralising antibodies against AAV capsid in serum (9/9 patients) and CSF (7/9 patients), capsid-specific T-cell responses in all patients that emerge 2–3 weeks post-injection, and upregulation of inflammatory chemokines (CXCL8, CXCL9, CXCL10) in CSF [154].

### 3.5. Neurotransmitter and Monoamine Disorder

#### Dopamine-Responsive Dystonia

Dopamine-responsive dystonia (DRD), also known as *Segawa syndrome*, is a rare genetic movement disorder primarily caused by mutations in genes involved in dopamine synthesis, such as guanosine triphosphate cyclohydrolase 1, *GCH1* (encoding GTP cyclohydrolase I), *TH* (tyrosine hydrolase), and *SR* (sepiapterin reductase) [155,156,157]. These mutations lead to dopamine deficiency in the nigrostriatal pathway, resulting in symptoms like dystonia, Parkinsonism, and diurnal fluctuation of symptoms [158]. Although the disease is highly treatable with levodopa therapy, some patients have been reported to experience incomplete responses or long-term complications [158].

Preclinical studies focus on using AAV serotypes with high tropism for dopaminergic neurons, such as AAV2, AAV9, and AAVrh10, for targeted gene therapy [159,160]. These serotypes enable efficient transduction of the striatum and substantia nigra, critical regions for dopamine synthesis. The goal is to either deliver GCH1 by restoring tetrahydrobiopterin (BH4) production or by seeking to directly enhance dopamine synthesis in striatal neurons by providing the TH gene [158,161], or a combination of both [160]. For example, AAV2-mediated delivery of GCH1 or TH genes in rodent models of DRD has demonstrated partial restoration of dopamine levels and improved motor function [160,161]. Currently, there are no publicly available data on a clinical trial exclusively for DRD; however, it will be interesting to see some of the preclinical studies translating to a human clinical trial in the coming years. DRD therapy may leverage strategies from Parkinson’s disease trials, such as intraparenchymal AAV2-GAD delivery to modulate neuronal circuitry [97,160,162].

## 4. Challenges

### 4.1. Species-Specific Barriers to CNS Tropism

The translation of laboratory research evidence to clinical applications remains a major hurdle today in combating several diseases. Data have shown that some AAV capsids that demonstrate effective transduction in the mouse CNS or brain have failed to demonstrate similar performance in other animal models. For example, AAV-PHP.B and related capsids show robust brain transduction in mice, but they fail to deliver similar efficiency in non-human primates and humans. The root cause is often the reliance on mouse-specific receptors like LY6A and LY6C1, which are absent or divergent in primate vasculature. Huang and colleagues engineered peptide-modified AAVs to bind LY6A/LY6C1 for mouse BBB crossing, then discovered that nearly all published “mouse-CNS” capsids exploit these proteins, rendering them ineffective in primates [163]. In an effort to address this problem, Ghauri and Ou reviewed how directed evolution and rational design can overcome such dependencies by selecting capsids on human cells or primate models rather than rodent brains [164]. Yet, translating capsid discoveries from rodents to primates remains a major bottleneck.

### 4.2. Cell-Type Specificity Within the CNS

Most wild-type serotypes (for example, AAV2 or AAV9) broadly transduce neurons and astrocytes, but actual targeting of microglia, oligodendrocytes, or distinct neuronal subtypes remains unclear. O’Carroll et al. note that promoter choice and serotype selection can modestly bias transduction toward astrocytes or oligodendrocytes, but no vector yet achieves exclusive, high-efficiency in vivo microglial transduction. This makes neurological diseases originating in these cells difficult to target [165].

Brown et al. deployed single-cell RNA sequencing to map AAV capsid tropism at cellular resolution, revealing that variants such as CAP-B10 preferentially infect glutamatergic neurons over astrocytes, and that subpopulations (Myoc+ astrocytes and pericytes) remain largely untargeted. These cell-type tropism maps underscore the need for continued capsid and promoter engineering tuned to proteomic and transcriptomic signatures of each CNS cell type [166].

### 4.3. Dynamic Tropism Under Physiological and Pathological States

To date, all AAV tropism data are derived from healthy animals. Yet, neuroinflammatory, BBB disruption or disease-specific brain tissue remodelling likely alter AAV distribution. Brown et al. observed that three days after systemic administration of AAV-PHP.eB, endothelial cells transiently upregulate p53 pathway genes. However, this acute response waned by day 25, suggesting that vascular stress reshapes cell susceptibility over time [166].

### 4.4. Delivery Method and Long-Term Expression Monitoring

In addition to capsid engineering for effective and efficient CNS tropism, another area of focus in AAV gene therapy for the CNS is therapeutic delivery strategies. The direct infusion of therapy into the putamen via stereotactic neurosurgery, for example Kebilidi^®^, is contraindicated in patients without radiographic evidence of skull maturity, excluding infants and many young children from treatment [167]. Preclinical rodent studies report stable transgene expression for 6–12 months, but long-term human data are sparse. Xu et al. found that while AAV delivers persistent gene expression in non-dividing neurons, suboptimal vector design and host immunity can erode expression over time, especially if the transgene provokes cytotoxic T cells or antibody responses.

Longitudinal analyses in non-human primates and multi-year follow-up of clinical cases (e.g., SMA gene therapy) suggest durability over 5 years; comprehensive datasets tracking transgene levels, immune markers, and functional outcomes beyond this window are lacking. Understanding the kinetics of vector genome silencing, immune clearance, or epigenetic regulation is critical for truly one-and-done CNS therapies. Secondly, it is unclear whether the therapeutic benefits persist beyond 2–3 years and provide long-term durability. Hence, there is a need for longitudinal follow-up studies.

What are the implications of tropism variability for re-dosing and immune responses? Do tropism changes directly or indirectly relate to disease stage or inflammation?

Every innovation comes with new challenges that must be carefully addressed. With new gene delivery methods gaining attraction, there is need to properly evaluate the long-term effects of these methods to ensure patients’ safety.

### 4.5. Off-Target Toxicity

Several AAV-based gene therapies have been shown to present off-target toxicity in different organs post-injection. Systemically (IV) delivered AAV9 and AAVrh10 penetrate the BBB, but their broad tropism also drives high ectopic expression in the liver, heart, and muscle. Ghauri and Ou document strategies to “de-target” peripheral organs, modifying surface loops or adding molecular “detours”, but stress that peripheral leakage remains problematic, contributing to hepatotoxicity and thrombocytopenia at high doses [164]. Daci and Flotte recently compared CSF, intraparenchymal, and IV delivery doses, showing that systemic administration requires vector loads ~10–100× higher than direct CNS injection, correlating with off-target organ exposures and immune activation [168]. Biology Insights further quantifies AAV9’s native affinity for hepatocytes and cardiomyocytes, cautioning that high IV doses induce dose-limiting toxicities in these tissues.

Several AAV-based gene therapies have been shown to present off-target toxicity in different organs post-injection. A recent study in *Signal Transduction and Targeted Therapy* used human-stem-cell-derived kidney organoids to model nephrotoxicity from AAV-mediated genome editing. Even without delivering a CRISPR/Cas9 payload, AAV2 alone triggered a robust NF-κB-dependent inflammatory cascade, leading to DNA damage, fibrotic remodelling, and cellular senescence [169]. After infecting the differentiated kidney organoid at 21 days with AAV serotypes with a multiplicity of infection (MOI) optimised by live fluorescence imaging, the data showed that AAV2 transduced ~46% of LTL^+^ proximal tubules and 46% of CDH1^+^ distal tubules, outperforming AAV8 and AAV9, which showed minimal uptake [169].

### 4.6. Failure to Meet the Expected Endpoint in Clinical Trials

Multiple AAV-mediated gene therapy clinical programmes have either failed to meet predefined efficacy endpoints or have been discontinued due to safety concerns, thereby highlighting critical translational limitations of this platform. In Parkinson’s disease, the AAV2-neurturin (CERE-120) trial did not demonstrate efficacy in a phase 2 study, as it failed to confer a clinically meaningful benefit, most likely due to suboptimal vector biodistribution and administration at relatively advanced stages of neurodegeneration [170,171]. Similarly, the CUPID2 trial evaluating AAV1–SERCA2a for the treatment of heart failure showed no improvement in clinical outcomes, with subsequent analyses implicating insufficient myocardial transduction and the negative impact of pre-existing neutralising antibodies [172]. In X-linked myotubular myopathy, high-dose systemic administration of AAV8–MTM1 (AT132) was associated with fatal hepatotoxicity, underscoring the risk of dose-dependent liver injury [173]. Likewise, systemic AAV micro-dystrophin gene transfer strategies for Duchenne muscular dystrophy have been associated with serious adverse events, including complement activation and acute hepatic injury, which highlights immune-mediated toxicities at higher vector doses [174]. Collectively, these outcomes have been attributed to suboptimal tissue transduction, host immune responses, dose-limiting toxicities, and challenges in translating preclinical efficacy to human subjects, as already discussed. These insights are now shaping contemporary approaches that emphasise advanced capsid engineering, targeted immune modulation, and optimised clinical trial design.

### 4.7. Cost and Access Barriers to AAV Gene Therapies in Low-Income Countries

**Figure 4:**
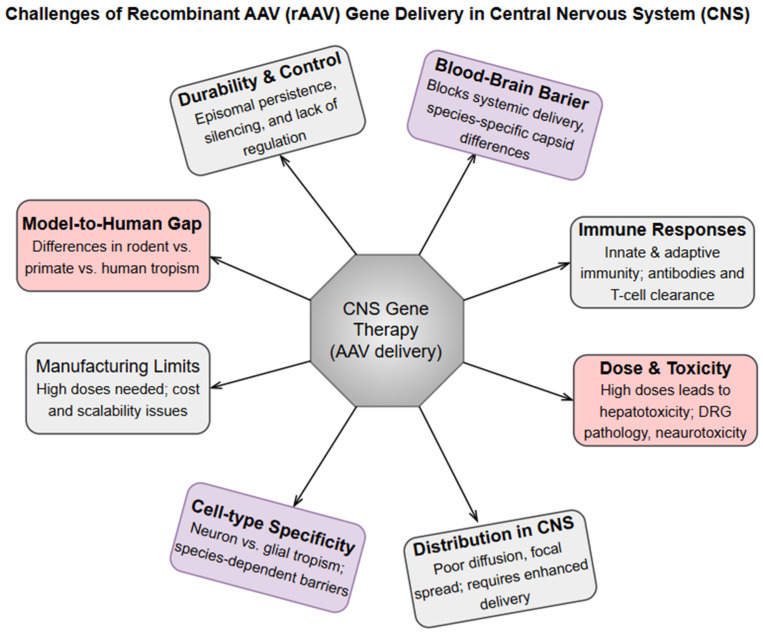
Schematic illustration of notable challenges in AAV gene therapy for neurological diseases.

The high prices of gene therapies and complex delivery requirements place AAV gene therapies out of reach for low-income countries. Manufacturing costs for clinical-grade vectors are enormous, requiring specialised facilities and rigorous quality control. The manufacturing complexity drives unit cost through expensive GMP facilities, high-value reagents, and low-yield viral vectors. In addition, technology transfer, local or indigenous capacity building, and partnerships (public–private and philanthropic) to ensure regional production and clinical deployment are a bottleneck [175,176]. See Figure 4 for other relevant challenges.

## 5. Future Perspectives

### 5.1. Capsid Engineering Strategies

**Figure 5:**
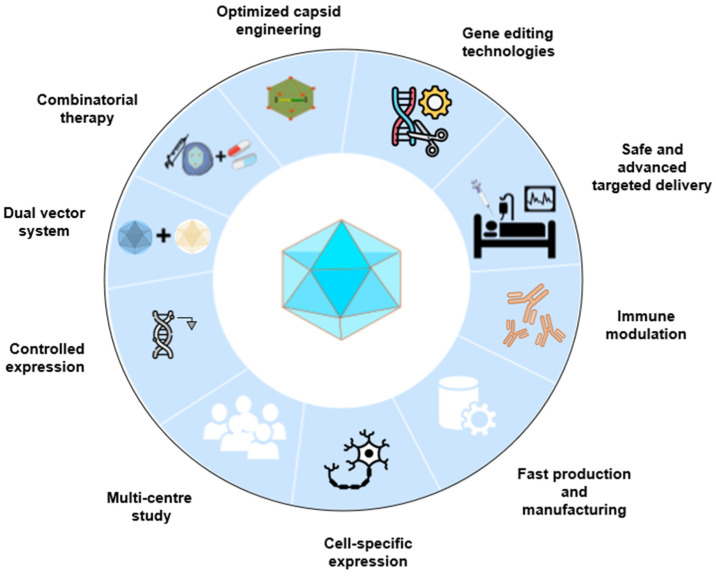
Summary of different approaches that transform the AAV gene therapy landscape to the CNS.

The future of AAV gene therapy for the CNS is likely to be defined by a combination of advances in capsid engineering, delivery strategies, payload design, and immunological control, all aimed at overcoming the intrinsic barriers that have limited the breadth and durability of clinical success. Although, current vectors have demonstrated meaningful efficacy in some cases, particularly through intrathecal or intraparenchymal delivery [177,178], the next generation of approaches will need to achieve efficient, widespread, and cell-specific transduction across the complex architecture of the human brain and spinal cord following minimally invasive administration (Figure 5).

The challenge of off-target and organ toxicity of AAV vectors is often the result of large doses of AAVs. The researchers at the CHOP recently developed novel AAV capsids with enhanced CNS tropism named AAV-Ep+ and AAV-DB-3. The AAV-Ep+ targets ventricular lining cells and cerebral neurons for lifelong enzyme secretion at lower doses, while AAV-DB-3 efficiently transduced deep brain and cortical structures in large animal models, reducing required vector doses significantly [179,180]. These innovations may address key limitations on potency and safety concerns, paving the way for one-time therapies. Also, there is a need for capsids that bind conserved human entry factors (e.g., AAVR/GPR108) and avoid reliance on species-restricted receptors like lymphocyte antigen 6 complex, locus A (Ly6a), using directed evolution, structure-guided design, and AI/ML to optimise tropism, immune evasion, and reduced ubiquitination [181].

Although several AAV serotypes and engineered variants have demonstrated relatively favourable biodistribution and transduction profile in the brain, their performance is highly dependent on both capsid properties and the route of administration. Efficient delivery to the CNS remains a central challenge in AAV gene therapy, owing to cellular complexity of neural tissues and the restrictive nature of the blood–brain barrier.

At the time of this review, White Lab Genomics announced at the American Society of Gene & Cell Therapy meeting in 2026 that their proprietary AI platform, ALFRED, enabled the design of a novel AAV vector with approximately 50-fold enhanced brain delivery compared to AAV9. This vector showed no detectable signal in the liver of wild-type mice and achieved functional targeting following standard intravenous administration (www.whitelabgx.com). Similarly, another AI-powered platform, REACH, showed promising results in AAV capsid engineering to penetrate the BBB in a non-human primate model with a 600–2000-fold increase in RNA expression level in the brain [182].

### 5.2. Cell-Specific Expression and mRNA De-Targeting

Equally important will be the refinement of AAV cellular specificity within the CNS. The ability to target neurons, astrocytes, oligodendrocytes, or microglia will be critical for addressing the diverse range of neurological disorders under investigation. Engineered vectors for controlled and inducible expression may be necessary for safety-first expression systems (inducible promoters, miRNA de-targeting, and protease-activatable switches) to avoid overexpression-related toxicity by resisting transgene expression to defined cell populations, even when transduction is widespread, and to enhance post-marketing modulation [164]. This dual-layer targeting approach may reduce off-target effects and improve therapeutic indices, particularly in diseases where aberrant expression in non-target cells could have deleterious consequences. Furthermore, addressing the dorsal root ganglion (DRG) and PNS neurotoxicity would be valuable for the future of AAV gene therapy for the CNS. Mechanistic studies and mitigation approaches (microRNA de-targeting, regulated expression, and dose optimisation) have been employed to reduce DRG pathology observed in NHPs after high-dose or intrathecal delivery, and to define biomarkers (e.g., NfL) for early detection [183,184].

### 5.3. Dual-Vector System

As technology advances, different approaches to AAV gene delivery to the brain continue to emerge. Examples include trans-splicing and the dual-vector approaches to delivering therapeutic genes, especially large genes. The hybrid approach was first described by Ghosh et al. in 2008 [185] and has been considered a promising approach that counters major concerns in the trans-splicing method [186]. Experimental data have shown the efficiency of this approach in delivering large transgenes in inherited retinal diseases [187,188,189,190,191] and muscle [192]. Researchers have also engineered advanced dual AAV platforms that exploit RNA trans-splicing and protein reconstitution strategies. For instance, Reidmayr et al. developed dual AAV vectors employing mRNA trans-splicing (REVeRT) that enable the reassembly of segmentally encoded, oversized expression cassettes at the mRNA level following co-transduction, thereby supporting functional expression of large or multiplexed modalities such as CRISPR-based transcriptional activators or base editors [193]. This strategy enhances flexibility in choosing split sites and improves the efficiency and robustness of functional reconstitution across co-delivered vector components.

Although the majority of these approaches remain at the preclinical stage, the overarching concept of combining distinct vector systems is being actively explored as a means to deliver larger genome-editing machineries or to coordinate multiple, independent therapeutic activities within the same cellular context, and this concept can be explored for CNS diseases as well. Although these strategies are considered promising, care must be taken to avoid immune activation, as every increase in viral load increases the chances of liver or other organ toxicity.

### 5.4. Combination Therapy

Pharmacological co-interventions also represent a promising avenue for enhancing AAV efficacy. Small molecules that modulate neuroinflammation, proteostasis, and cellular stress response can improve neuronal survival and transgene performance. Importantly, immunomodulatory regimens, including corticosteroids and other immunosuppressive agents such as prednisolone, methylprednisolone, and prednisone have been used clinically to mitigate immune responses to AAV capsids and transgene products, thereby improving safety and persistence of expression [194,195,196]. Recently, Wave Life Sciences demonstrated how combination therapy could be a way forward. They developed WVE-004, a gene-silencing therapy targeting C90rf72, and ION363, an Ionis therapy targeting FUS, both of which hold promise for future clinical translation. Both therapies incorporate antisense oligonucleotide (ASO) and are currently in phase II and III trials, respectively [104]. Hence, designing AAV vectors that incorporate this nucleotide may provide therapeutic benefits for millions of patients globally. Another area of interest would be the use of organoids to model CNS organs [197]. Humanised xenografts, organoids, and NHPs with human receptor expression (or receptor knock-ins) can be used to reduce mouse-to-human translational failures and to test immune/DRG safety in a clinically relevant context [181].

Additionally, from a systems perspective, combination therapies acknowledge that many CNS disorders involve complex interactions between neurons, glial cells, and immune pathways, and AAV-mediated delivery of a single gene may not be sufficient to address this complexity or produce gene expression capable of restoring normal functioning. By simultaneously targeting multiple pathways or cell types, combination approaches could produce synergistic effects and improve functional recovery. This is particularly relevant in neurodegenerative diseases, where both neuroprotection and regeneration may be required for meaningful clinical benefit.

For the TREM2 target in AD, most published TREM2 overexpression studies in Alzheimer’s disease models have actually used lentiviral or transgenic approaches rather than AAV. In APPswe/PS1dE9 mice, upregulated TREM2 was achieved by using lentiviral vectors for delivery into the brain parenchyma. For example, Ruganzu et al. used a lentiviral construct carrying TREM2 to overexpress the receptor in the APP/PS1 hippocampus and reported reduced amyloid deposition, decreased neuroinflammation via JAK/STAT/SOCS signalling, and improved cognition [198]. Jiang et al. similarly employed lentiviral vectors for TREM2 (overexpression and knockdown) in primary microglia and in APP/PS1 mouse brains, showing that increased TREM2 enhanced Aβ phagocytosis and reduced Aβ-induced pro-inflammatory responses [199]. The human TREM2 cDNA is approximately 820 base pairs long and encodes a 230-amino acid type 1 transmembrane glycoprotein with a theoretical molecular weight (MW) of 25.4 kDa (Uniprot: Q9NZC2) [200,201,202]. A minimal AAV expression cassette for TREM2—including a CNS- or microglia-active promoter (such as CX3CR1, CD68, or short synthetic promoters around 200–400 bp), the TREM2 transgene, a polyA signal (SV40 or bGH, ~200 bp), and ITRs—fits comfortably within the standard AAV packaging limit of 4.7 kb ssDNA. Even with added regulatory elements like miRNA detargeting for neuron sparing (~100–200 bp) or a WPRE for enhanced expression (~600 bp), the total cassette size would remain under 4 kb, leaving ample headroom and avoiding the inefficiencies or truncations seen with oversized payloads above 5.2 kb [203]. For future work, this gap defines a clear opportunity. However, designing an AAV-TREM2 system will require making first-principles choices about capsid (for microglial versus general CNS targeting), promoter strength and specificity, and dosing window, rather than simply borrowing a “standard” AAV-TREM2 vector system.

### 5.5. Improved Large-Scale Manufacturing Process

From a translational perspective, improvement in manufacturing, vector quality, and dose standardisation will be essential to support broader clinical applications of AAV gene therapy. Across all manufacturing platforms, downstream processing of rAAV remains a critical determinant of vector quality [204,205]. Purification methods such as caesium chloride or iodixanol density gradient ultracentrifugation are commonly used at laboratory scale to separate full from empty capsids, but these methods are difficult to scale and are labour-intensive. More recently, tangential flow filtration (TFF), and mass photometry (MP) techniques were developed as alternative methods for fast, efficient and high-quality AAV viral purification [206,207]. On the other hand, chromatography-based approaches, including ion exchange and affinity chromatography, have been developed for large-scale purification and offer improved scalability and reproducibility [208,209,210]. Although MP techniques present some advantages over ion exchange and affinity chromatography such as the ability to resolve and quantify not only empty and full capsid populations but also to identify partially packaged capsid impurities [206], the scalability of such techniques at industrial scale may present another layer of challenge. These manufacturing bottlenecks add to the problems of vector quality, quantity and cost.

The integration of high-resolution chromatography techniques and novel affinity ligands capable of discriminating between empty and full capsids could transform the AAV purification process.

Despite increasing knowledge of AAV biology, the relationships between vector dose, biodistribution, effect of empty capsid and toxicity remain incompletely understood, particularly in the context of systemic delivery to the CNS.

**Figure 6:**
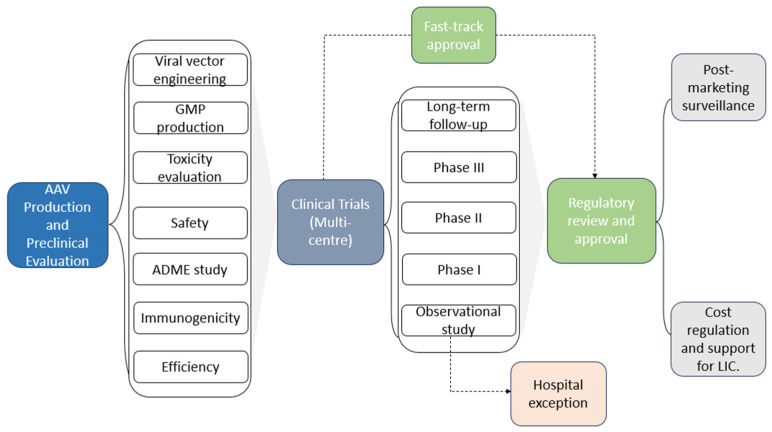
Schematic illustration of the process of AAV production cycle, clinical trial and regulatory approval. ADME (adsorbtion, distribution, metabolism, excretion)—the four core stages of pharmacokinetics—absorption, distribution, metabolism, and excretion; GMP—good manufacturing practice; LIC—low-income countries.

Finally, there is a need for parallel investment in decentralised delivery infrastructure, cost-reducing manufacturing, and payer engagement so that AAV CNS therapies can move beyond single-centre, ultra-high-cost treatment to multicentre clinical trials that take into account the genetic diversity across the global population that might hinder therapeutic efficacy and broad clinical usage [211]. In Figure 6, we summarised the process of the AAV production cycle, clinical evaluation and regulatory approval, which might increase the efficacy and validity of AAV gene therapy to the CNS.

## 6. Conclusions

The field of AAV gene therapy for genetic diseases of the central nervous system has evolved significantly from first-generation approaches to advanced vector engineering strategies that enhance safety, efficacy, and accessibility. Next-generation promoters, such as the hybrid synapsin/CMV system, for example in EXG001-307, enable tissue-preferential expression, thereby expanding the therapeutic window. Novel vector technologies such as cceAAV offer improved manufacturing consistency and transduction efficiency. There is no doubt that further advances in protein language models (pLMs), combined with machine learning paradigms such as reinforcement learning (RL), will enable the engineering of novel, enhanced AAV capsids for precise delivery to targeted brain cells. Alternative delivery routes, especially intrathecal administration and CSF delivery, extend treatment potential to older patients while reducing systemic toxicity.

Despite these advances, significant challenges remain regarding optimal time of intervention, tissue-specific expression control to reduce off-target and organ toxicity, and global access to these transformative therapies. The development of cost-effective alternatives for resource-limited, low-income, and developing countries, and the exploration of combination therapies addressing both dependent and independent pathways, will represent promising future directions. The Global Gene Therapy Initiative (GGTI) and other stakeholders must prioritise regulating tiered pricing linked to country income levels and outcomes-based payment models that spread cost over time, conditional on patient benefit, and ensuring technology transfer and capacity building partnerships to establish regional GMP vector manufacturing hubs that lower unit cost via scale and proximity.

As the field continues to evolve, ongoing research must focus not only on technological innovations but also on implementation strategies that ensure the equitable distribution of these life-changing therapies to all sufferers of these debilitating disorders worldwide. The remarkable progress in AAV gene therapy for CNS disorders serves as a paradigm shift for the treatment of monogenic neurological disorders and offers hope for increasingly effective and accessible treatments in the coming years.

## Figures and Tables

**Table 1:** Comparison of transduction efficiency of natural AAV capsids for CNS/brain delivery with respect to injection routes.

Serotype	Primary Receptor	IV Efficiency	IT/ICV Efficiency	Primary Cell Target	Species Efficiency
AAV1	Sialic acid, FGFR1	Very low	Moderate	Neurons (cortex, spinal cord), astrocytes	It demonstrates efficient motor neuron transduction after intrathecal delivery in NHP [22]
AAV2	HSPG, FGFR1, αVβ5 integrin	None in primates	Moderate	Neurons (striatum, hippocampus), ependymal cells	It has broad transduction in human brain organotypic slices; used in Kebilidi^®^ (intracranial) and Parkinson’s trials (AAV2-GAD) [39,40]
AAV4	Sialic acid	Unknown	Moderate	Neurons, ependymal cells	Ability to transduce NHPs/human cells
AAV5	PDGFR, sialic acid	Low	High	Astrocytes, ependymal cells, and cortical neurons	It shows robust transduction in NHP cortex/striatum through ICV delivery; used in Huntington’s trials [21,40]
AAV6	α2,3-/α2,6-linked sialic acid, EGFR, HSPG	NA	NA	Neurons (hippocampus, striatum, cortex)	The vector demonstrates high transduction efficiency in NHPs across different sections of the brain
AAV7	NA	Moderate (liver bias)	Low	Limited neurons, microglia	Better efficient tropism in skeletal muscle; demonstrates weak CNS transduction via IT delivery in NHPs [41]
AAV8	LamR	Very low	Moderate–high	Purkinje cells, spinal motor neurons	Efficient spinal cord transduction through IT delivery in NHPs [22]
AAV9	Galactose, LamR, AAVR	Low	High	Neurons, astrocytes, and microglia	Crosses BBB in infants, evident in Zolgensma^®^; broad NHP transduction via IT [20]
AAV11	Unknown	Low	Moderate	Neurons (thalamus), endothelial cells	Cortical transduction after ICM injection route
AAVrh.10	Unknown	Low	High	Neurons (cortex, striatum)	Used in GM2 gangliosidosis and Alzheimer’s trials [21,40]

AAV: adeno-associated virus; AAVR: adeno-associated receptor; CNS: central nervous system; EGFR: epidermal growth factor receptor; FGFR1: fibroblast growth factor receptor 1; HSPG: heparan sulfate proteoglycan; IV: intravenous; IT: intrathecal; ICV: intracerebroventricular; ICM: intra-cisterna magna; LamR: laminin receptor; NHP: non-human primate; PDGFR: platelet-derived growth factor receptor.

**Table 2:** Core performance metrics: natural AAV capsids versus recombinant AAVs.

Feature	Natural AAV Capsid	Recombinant/Engineered AAV Capsid	Superiority of Recombinant over Natural AAV
BBB Penetration	Poor in adults (AAV9: <0.1% IV dose reaches brain	Over 50-fold improvement in transduction (for example, AAV-PHP.B and AAV-CAP.B10) via IV	Enables non-invasive systemic dosing for CNS disorders [49,50]
Cell-Type Specificity	Broad tropism (for example, AAV9 transduces neurons and glia)	Cell-restricted expression (for example, AAV-MG1.1 for microglia)	Reduces off-target effects; supports precision and personalised therapy [51]
Immunogenicity	High pre-existing Nabs in humans (30–70% in AAV2/9)	Escapes NAbs (for example, AAV-TT and AAV-LK03 vectors)	Provides opportunity for more treatable patients; enables redosing [52,53]
Tropism Flexibility	Fixed receptor binding (HSPG in AAV2; LamR in AAV8)	Retargeting receptor-binding (for example, TfR1-binding peptides)	Allows for targeting of resistant tissues (blood vessels, for example) [54]

**Table 3:** Selected ongoing clinical trials for AAV gene therapy in different CNS disorders.

Company/ Institution	Disease Condition	Therapy	AAV Vector	Delivery Route	Phase
Novartis	Spinal muscular atrophy (SMA)	Zolgensma^®^	AAV9	Intravenous	FDA-approved
PTC Therapeutics	Aromatic L-amino acid decarboxylase (AADC) deficiency	Upstaza^®^	AAV2	Intracranial	EMA-approved
		Kebilidi^®^	AAV2	Intracranial	FDA-approved
Lexeo Therapeutics	Alzheimer’s disease (AD)	AAVrh10hAPOE2 (LX1001) encoding human apolipoprotein E2	AAVrh.10	Intrathecal	Phase 1/2 (NCT03634007)
University of California	Alzheimer’s disease (AD)	AAV2-BDNF (Brain-Derived Neurotrophic Factor)	AAV2	Stereotaxically MRI-guided injection into the brain	Phase 1 (NCT05040217)
AviadoBio Ltd.	Frontotemporal Dementia with Progranulin Mutations (FTD-GRN)	AVB-101 (AAV.PGRN)	AAV9	Intrathalamic administration	Phase 1/2 (NCT06064890)
Brainvectis, a subsidiary of AskBio Inc.	Huntington’s disease	AB-1001 (AAVrh10.CAG.hCYP46A1)	AAVrh.10	Intracerebral injection into the striatum	Phase 1/2 (NCT05541627)
UniQure Biopharma B.V.	Huntington’s disease	AMT-130 (rAAV5-miHTT)	rAAV5	Intrastriatal MRI-guided injection	Phase 1/2 (NCT04120493)
Spark Therapeutics, Inc.	Huntington’s disease	SPK-10001	Not disclosed	Intraparenchymal infusion	Phase 1/2 (NCT06826612)
Lantu Biopharma	Spinal muscular atrophy (SMA)	LT01-101 (AAV-hSMN1	AAV9	Undisclosed	Phase 1/2 (NCT06288230)
GeneCradle Inc	Spinal muscular atrophy (SMA) types 1, 2, and 3	GC101 self-complementary recombinant adeno-associated viral vector (scAAV) containing a single-stranded transgene encoding a codon-optimised human SMN1 gene	AAV9	Intrathecal (IT) injection	Phase 3 NCT05824169, NCT06421831, NCT06971094
Novartis Gene Therapies	Spinal muscular atrophy (SMA) type 1	OAV101 (AVX-101-Onasemnogene abeparvovec-xioi in spinal muscular atrophy (SMA) type 1)	AAV9	Intravenous injection	Phase 3 NCT03306277, NCT05089656

## Data Availability

No new data were created or analyzed in this study. Data sharing is not applicable to this article.
